# The epidemiology of patients with toxoplasmosis and its associated risk factors in Taiwan during the 2007–2020 period

**DOI:** 10.1371/journal.pone.0290769

**Published:** 2023-08-25

**Authors:** Chia-Peng Yu, Bao-Chung Chen, Yu-Ching Chou, Chi-Jeng Hsieh, Fu-Huang Lin

**Affiliations:** 1 School of Public Health, National Defense Medical Center, Taipei City, Taiwan; 2 Division of Gastroenterology, Department of Internal Medicine, Tri-Service General Hospital, National Defense Medical Center, Taipei City, Taiwan; 3 Department of Health Care Administration, Asia Eastern University of Science and Technology, New Taipei City, Taiwan; Universite Ziane Achour - Djelfa, ALGERIA

## Abstract

**Background:**

Toxoplasmosis is a zoonotic parasitic disease caused by the protozoan *Toxoplasma gondii (T*. *gondii)*, and may cause miscarriage and birth defects during pregnancy. This study aimed to assess the epidemiological features, epidemic trends, and correlations between the before number of confirmed toxoplasmosis cases in Taiwan from 2007 to 2020 in gender, age, season, and residential area, and hypothesized the environmental and climate factors also might affect the disease in Taiwan.

**Methods:**

This study reviewed publicly available annual summary data on reported toxoplasmosis cases in the Taiwan Centers for Diseases Control (TCDC) between 2007 and 2020.

**Results:**

This study collected 150 confirmed domestic and nine patients with imported toxoplasmosis. There was an increasing trend in the incidence of toxoplasmosis, 0.09–0.89 cases per 1,000,000 people, peaking in 2017. The average annual toxoplasmosis incidence was 4.4, 13, and 18 during 2007–2011, 2012–2016, and 2017–2020, respectively. Comparing sex, age, season, and place of residence, the incidence rate was highest in male, 20–39 years-old patients, summer, and the eastern region, with 1.02, 1.72, 0.38, and 3.63 cases per million population, respectively. Additionally, comparing the distribution of cases by age group in Taiwan, there were significant differences between 40–59 years-old in the northern region (odds ratio (OR) = 0.343, 95% confidence interval (CI) = 0.167–0.705, *p* = 0.004) and 40–59 years-old in the southern region (OR = 4.488, and 95% CI = 2.149–9.374, *p*< 0.001), respectively. Linear regression analysis also showed that PM (particulate matter) 2.5 (μg/m^3^) concentration was positively associated with toxoplasmosis (β = 0.095, *p* = 0.037). CO concentration was negatively correlated with toxoplasmosis (β = -14.001, *p* = 0.021).

**Conclusions:**

This study is the first to report domestic and confirmed cases of imported toxoplasmosis from the surveillance data of the TCDC between 2007 and 2020. It identified that residence and age were associated with an increased risk of toxoplasmosis in Taiwan. This study confirmed that toxoplasmosis remains a prevalent infectious disease in Taiwan, its epidemic is gradually increasing and becoming more severe. These findings might be useful for policy-makers and clinical experts to direct prevention and control activities to patients with *T*. *gondii*, which causes the most severe illness and greatest burden to Taiwanese people.

## Introduction

Toxoplasmosis is a zoonotic infectious disease caused by *T*. *gondii* which infects approximately one-third of the world population among humans [1–3]. An estimated 8–22% of the people in the USA are infected, with a similar prevalence rate in the UK [4–8]. In Europe, South America, and Central America, estimates of infection ranged between 30 and 90% [5, 9–11]. A previous study has shown that toxoplasmosis was the second most common cause of death attributable to food-borne diseases (estimated 327 deaths) and the fourth leading cause of hospitalizations due to food-borne illnesses (4428 hospitalizations) from the mid-to-late 2000s in the USA [12–14]. *T*. *gondii* is an intracellular protozoa that infects warm-blooded animals. The intermediate hosts of *T*. *gondii* are birds and mammals, including cattle, rodents, sheep, and humans, with cats as the definitive hosts [15]. Sexual reproduction only occurs in cats, where in the epithelial cells of the small intestine, the female and male gametes combine to form oocysts that are excreted in cat feces. Oocysts are infective after 24–48 h of sporulation. After ingestion by warm-blooded animals, sporulated oocysts enter epithelial cells and nearby lymphoid tissues, divide into tachyzoites, and form tissue cysts in the brain, retina, striated muscle, and liver cells. There are hundreds of bradyzoites in tissue cysts, which are transmitted to people or animals that eat raw or undercooked meat. Oocysts are tolerant to environmentally stressful conditions and can survive for more than 1 year under appropriate conditions. However, these oocysts are killed after boiling for 5 min at 77°C. The transmission potential of tachyzoites is very low compared with that of tissue cysts (bradyzoites) and oocysts [16].

Human infections with *T*. *gondii* can be divided into congenital and acquired ones. Congenital infections result from *T*. *gondii* infections during pregnancy. *T*. *gondii* is then transmitted to the fetus through the placenta [2, 17]. Acquired infections are mainly caused by eating raw food, undercooked infected animal meat (or body fluid), containing tissue cysts, such as those from cattle, sheep, and pork. Cat feces may contain oocysts of *T*. *gondii*, which contaminate soil and water. Rare instances of transmission are caused by receiving infected blood via transfusion during organ transplantation [18–20].

A few patients with normal immune function exhibited obvious symptoms, the most common of which were lymphadenopathy and fatigue. If pregnant women are infected, there is a risk of fetal infection. If fetus develops brain infection, there may be sequelae, such as hydrocephalus, cerebellar disease, retinochoroiditis, and epileptic seizures [2, 17]. A severely infected fetus may be stillborn or die within days of birth. A few months after congenital *T*. *gondii* infection, newborns experience poor vision, learning disabilities, and mental retardation [21]. In addition, the symptoms in immunocompromised patients, such as those with human immunodeficiency virus (HIV), patients with cancer receiving immunosuppressive therapy, or organ transplant recipients, are often caused by *T*. *gondii* reactivation from previous infections. The disease burden of *T*. *gondii* and possibility of disease outbreaks highlight the importance of identifying risk factors related to this disease [22]. Environmental factors are closely related to the infection rate of food-borne diseases [23], such as toxoplasmosis.

Taiwan is located at latitude 23°4’ north and longitude 121°0’ east and has a subtropical climate. The average monthly temperature ranges between 16°C and 29°C, and the average monthly relative humidity ranges between 75% and 90%. Taiwan has become a developed country with a per capita gross domestic product (GDP) of US$27,437 [24]. Although effective treatment methods are available, cases of toxoplasmosis infection still exist in Taiwan, indicating that epidemic prevention measures to eliminate the disease are insufficient [25]. Local cases of toxoplasmosis occur year-round in Taiwan, and the number of cases of imported infections reported abroad is small. However, there is little epidemiological information on the risk of toxoplasmosis in Taiwan using big data [25]. To bridge this knowledge gap, this study aimed to use data from the Taiwan National Infectious Disease Statistics System (TNIDSS) to evaluate the prevalence of toxoplasmosis in the Taiwanese population according to sex, age, season, and area of residence from 2007 to 2020. The correlation between disease characteristics, epidemic trends, environmental factors, and number of cases will be elucidated.

## Materials and methods

### 2.1. Ethical statement

The data was collected from the Taiwan Centers for Disease and Control (TCDC), which is a public health surveillance rather than research involving human participants. All data were de-identified in this study, and we ensured that all data were fully anonymized before we accessed them. Thus, the institutional review board approval and informed consent were not required for deidentified secondary data analyses [26].

### 2.2. Definition of confirmed cases

#### 2.2.1. Clinical conditions

First, congenital infection included newborns who had suspected *T*. *gondii* infection after birth, including clinical manifestations such as brain calcification, hydrocephalus, cerebellum, retinochoroiditis, glaucoma, pneumonia, myocarditis, hepatosplenomegaly, rash, neonatal jaundice, and other symptoms. Second, regarding acquired infections: Most were asymptomatic, and only 10–20% of patients would have swollen lymph nodes or flu-like symptoms in the acute stage. Individuals with a deficient immune system may experience clinical manifestations, such as retinochoroiditis, pneumonia, pericarditis, myocarditis, or encephalitis.

#### 2.2.2. Test conditions

Test conditions included any of the following: First, *T*. *gondii* was found in tissue section. Second, clinical samples (serum or cerebrospinal fluid) of *T*. *gondii* were isolated using bioassays or cell culture. Third, clinical samples tested positive based on PCR. Fourth, Serological testing for antibody positivity: IgM antibody against *T*. *gondii* was positive in paired serum.

A notified case was defined as that having any of the following conditions: (i) case that met the clinical conditions; (ii) patient who was diagnosed with toxoplasmosis by a physician, where in specimen was clinically tested and met the test conditions. A confirmed case was defined as patient who met both the clinical and laboratory conditions.

Physicians could also use epidemiological tools for the auxiliary diagnosis of toxoplasmosis. Epidemiological tools included to investigate the case have a history of any of the following characteristics: Contact with cats and ingestion of undercooked animal meat, tissue, body fluids or food or water contaminated. These investigation also comprised vertical infection of mother and child and infection caused by blood transfusion and organ transplantation.

### 2.3. Data source

Taiwan has a population density of 627/km^2^, area of 36,188 km^2^, and population of approximately 23.5 million. The majority (95%) of the population lives in the western part of Taiwan and are divided into the northern, central, and southern regions. Only 5% of Taiwan’s population lives in eastern Taiwan, where healthcare and socioeconomic status are classified as vulnerable. In addition, there are three outlying islands: Penghu, Kinmen, and Matsu.

Three databases were used for this study. The first was the TNIDSS, which is a publicly available Internet database on the TCDC website [27]. This database includes all statutory infectious diseases in categories 1–5, as stipulated by the Law on Prevention and Control of Infectious Diseases. To ensure information security and prevent privacy leakage, the database of the system did not store any case details and only included secondary data of statistical values. Since 2007, the TCDC has created the Toxoplasmosis Infectious Disease Statistical Data Query System to provide the public, academia, and media with the latest information on toxoplasmosis, which is classified as the fourth category of notifiable infectious diseases under the "Infectious Disease Prevention and Control Law". The data that can be accessed openly included the number of confirmed cases of toxoplasmosis imported from January 2007 to December 2020. Internet databases included sex, age, season, and residence related to cases of toxoplasmosis. Internet databases do not contain the medical history of patients, signs and symptoms, or treatment. In addition, this study analyzed monthly air pollutant data provided by the Taiwan Environmental Protection Agency (EPA) air quality monitoring network from 2013 to 2020 [28] (total suspended particulates [TSP], fine suspended particulates [PM2.5], nitrogen dioxide [NO2], sulfur dioxide [SO2], carbon monoxide [CO], and ozone [O3]. Monthly data on weather factors (temperature, rainfall, relative humidity, atmospheric pressure, rainfall days, and sunshine hours) from 2009 to 2020 were provided by the Taiwan Meteorological Bureau of the Ministry of Communications [29]. Statistical analysis and correlation tests were used to understand the temporal and spatial variation trends of air pollutants, meteorological factors, and their correlation with the number of *T*. *gondii* cases.

### 2.4. Statistical analyses

This study determined the number of native and cases of imported toxoplasmosis from a database from 2007 to 2020 and reviewed the distribution of epidemiological characteristics (sex, age, date of diagnosis, season, and place of residence); differences; and outcomes. Descriptive data are shown as means and summary statistics, where appropriate. Categorical variables were compared using the chi-squared test. This study computed the odds ratio (OR) using logistic regression and 95% confidence interval (CI) for parameter estimation. Environmental variables that were significantly correlated with toxoplasmosis were tested for independence using multiple linear regression analysis. All statistical analyses were performed using the SPSS software (IBM SPSS Statistics 21; Asia Analytics Taiwan Ltd., Taipei, Taiwan). All statistical tests were 2-sided with an α level of 0.05. Statistical significance was set at *p*< 0.05.

## Results

### 3.1. Distribution of confirmed cases in terms of sex, age, season, and place of residence during 2007–2020

There were 159 confirmed cases of local and imported infection, 150 confirmed local cases (94.3%), 81 male patients (54.0%), 71 cases between the ages of 20–39 years-old (47.3%), 49 cases in summer (32.7%), and 62 cases in the northern region (41.3%). There were nine confirmed cases of imported infection (5.7%), including six female patients (66.7%), six cases between the ages of 20–39 years-old (66.7%), four cases in the summer (44.4%), and five people living in the northern region (55.6%), which had the highest proportion (Table 1).

**Table 1:** Demographic characteristics of toxoplasmosis in Taiwan, during 2007–2020 period.

Variables	categories	Domestic cases (n = 150)	Imported cases (n = 9)	*P*
Gender				
	Male	81	3	0.228
	Female	69	6	
Age group (years-old)				
	<20	14	0	0.585
	20–39	71	6	
	40–59	52	2	
	≧60	13	1	
Season				
	Spring	31	0	0.489
	Summer	49	4	
	Fall	32	2	
	Winter	38	3	
Region of residence				
	Northern	62	5	0.615
	Central	35	1	
	Southern	41	3	
	Eastern	12	0	

### 3.2. Comparison between the numbers of confirmed cases each year in relation to differences in identity, sex, age, season, and place of residence

The number of local confirmed cases was 21 in 2017. Moreover, the numbers of confirmed cases of imported infection were four in 2019, which accounted for the highest proportion (14.0% versus 44.4%). The number of confirmed cases in male patients was 59 during 2014–2020 (54.6%). The number of confirmed cases in women during 2007–2013 was 26 (51.0%). The number of confirmed cases in those aged 20–39 years-old was 25 in 2007–2013, and 52 in 2014–2020. The number of confirmed cases in the winter and summer was 17 during 2007–2013 and 39 during 2014–2020, respectively. The number of confirmed cases in the northern region was 22 during 2007–2013, and 45 during 2014–2020. There was no statistically significant difference between the number of confirmed cases during 2007–2013 and 2014–2020, and the relationship between different identities (local or imported), sex, age group, season, and place of residence (all *p*>0.05) (Table 2).

**Table 2:** Epidemiological features of toxoplasmosis in Taiwan during 2007–2020 period.

Variables	Categories	Year		*p*
		2007–2013	2014–2020	
imported cases				
	Yes	3	6	0.934
	No	48	102	
Sex				
	Male	25	59	0.508
	Female	26	49	
Age group				
	<20	8	6	0.167
	20–39	25	52	
	40–59	14	40	
	≧60	4	10	
Season				
	Spring	11	20	0.377
	Summer	14	39	
	Fall	9	25	
	Winter	17	24	
Region of residence				
	Northern	22	45	0.859
	Central	13	23	
	Southern	12	32	
	Eastern	4	8	

### 3.3. Comparison between the number of confirmed cases in relation to gender, age group, and place of residence by seasons

There were 19 female patients in the spring. There were 27, 21, and 24 male patients in the summer, autumn, and winter, respectively. The number of confirmed cases in the spring, summer, autumn, and winter in the 20–39 years-old age group were 16, 26, 15, and 20, respectively. There were 17, 22, 13, and 15 patients living in the northern region from the spring to winter. There was no statistically significant difference between the number of confirmed diseases in different sexes, age groups, or places of residence by season (all *p*>0.05) (Table 3).

**Table 3:** Epidemiological features of seasonality of toxoplasmosis in Taiwan during 2007–2020 period.

Variables	Categories	Season				
		Spring (n = 31)	Summer (n = 53)	Fall (n = 34)	Winter (n = 41)	*P*
Sex						
	Male	12	27	21	24	0.243
	Female	19	26	13	17	
Age group						
	<20	4	4	2	4	0.950
	20–39	16	26	15	20	
	40–59	8	20	13	13	
	≧60	3	3	4	4	
Region of residence						
	Northern	17	22	13	15	0.325
	Central	3	13	9	11	
	Southern	8	16	11	9	
	Eastern	3	2	1	6	

### 3.4. Comparison between confirmed cases of toxoplasmosis in relation to gender, age and place of residence

The number of confirmed diseases in the age group of 30–39 years-old was 40 and 37 in male and female patients, respectively. The number of confirmed cases in the northern region was 28 in male and 39 in female patients. There was no statistically significant difference in the relationship between the number of confirmed diseases and sex, age group, and place of residence (all *p*>0.05) (Table 4).

**Table 4:** Epidemiological features of toxoplasmosis in Taiwan in relation to gender during 2007–2020 period.

Variables	Categories	Sex		*p*
		Male (n = 84)	Female (n = 75)	
Age group (years-old)				
	<20	7	7	0.781
	20–39	40	37	
	40–59	31	23	
	≧60	6	8	
Region of residence				
	Northern	28	39	0.053
	Central	23	13	
	Southern	28	6	
	Eastern	5	7	

### 3.5. Comparison between confirmed cases of toxoplasmosis in relation to place of residence in different age groups

The number of confirmed cases in the northern region was 8, 36, and 9 in the <20, 20–29, and ≥60 years-old age groups, respectively. The number of confirmed cases in the southern region was 26 in the 40–59 age group. The OR for confirmed cases in the northern region compared with other regions was 0.343 (95% CI = 0.167–0.705). The number of confirmed cases in the southern region in the age groups of 20–39 and 40–59 years-old compared with other regions was OR = 0.385 (95% CI = 0.185–0.802), while the number of confirmed cases in the southern region compared with other regions was OR = 4.488 (95% CI = 2.149–9.374) (Table 5).

**Table 5:** Epidemiological features of toxoplasmosis in Taiwan in relation to age group during 2007–2020 period.

Variables	categories	Age group				*p*
		<20 (n = 14)	20–39 (n = 77)	40–59 (n = 54)	≧60 (n = 14)	
Region of residence						
	Northern	8	36	14^a^	9	0.009
	Central	3	21	9	3	
	Southern	3	14^b^	26^c^	1	
	Eastern	0	6	5	1	

^a^: OR = 0.343, 95% CI = 0.167–0.705 (*P* = 0.004)

^b^: OR = 0.385, 95% CI = 0.185–0.802 (*P* = 0.011)

^c^: OR = 4.488, 95% CI = 2.149–9.374 (*P*< 0.001)

### 3.6. The relationship between multiple environmental predictors and the number of toxoplasmosis cases

Linear regression analysis showed that PM (2.5) concentration was associated with toxoplasmosis (β = 0.095, standard error = 0.045, *p* = 0.037) (Table 6). Linear regression analysis also showed that the CO concentration and toxoplasmosis cases were associated (β = -14.001, standard error = 5.937, *p* = 0.021) (Table 6). Linear regression analysis showed that climatic factors were associated with toxoplasmosis cases (R2 = 0.037, F = 0.877, *p* = 0.513) (Table 7).

**Table 6:** Association between air pollution factors and toxoplasmosis cases by multiple linear regression analysis.

Variables	Non-standardization coefficient		*p*
	β value	Standard error	
TSP (μg/m^3^)	-0.012	0.014	0.395
PM 2.5 (μg/m^3^)	0.095	0.045	0.037
SO_2_ (ppb)	-0.262	0.283	0.356
CO (ppm)	-14.001	5.937	0.021
NO_2_ (ppb)	0.211	0.172	0.225
O_3_ (ppb)	-0.027	0.017	0.127

R^2^ = 0.140; F = 2.424 (*P* value: 0.032); df = (6, 89)

N = 96

**Table 7:** Association between climate factors and toxoplasmosis cases by multiple linear regression analysis.

Variables	Non-standardization coefficient		*p*
	β value	Standard error	
Temperature (°C)	0.002	0.061	0.974
Precipitation (mm)	<0.001	0.001	0.873
Relative humidity (%)	0.088	0.052	0.090
Mean Pressure (hPa)	0.040	0.049	0.416
Number of Days with Precipitation ≧0.1mm (day)	-0.029	0.063	0.642
Sunshine Duration (hr)	0.005	0.005	0.302

R^2^ = 0.037; F = 0.877 (*P* value: 0.513); df = (6, 137)

N = 144

### 3.7. Trend of the incidence of confirmed cases

**Fig 1:**
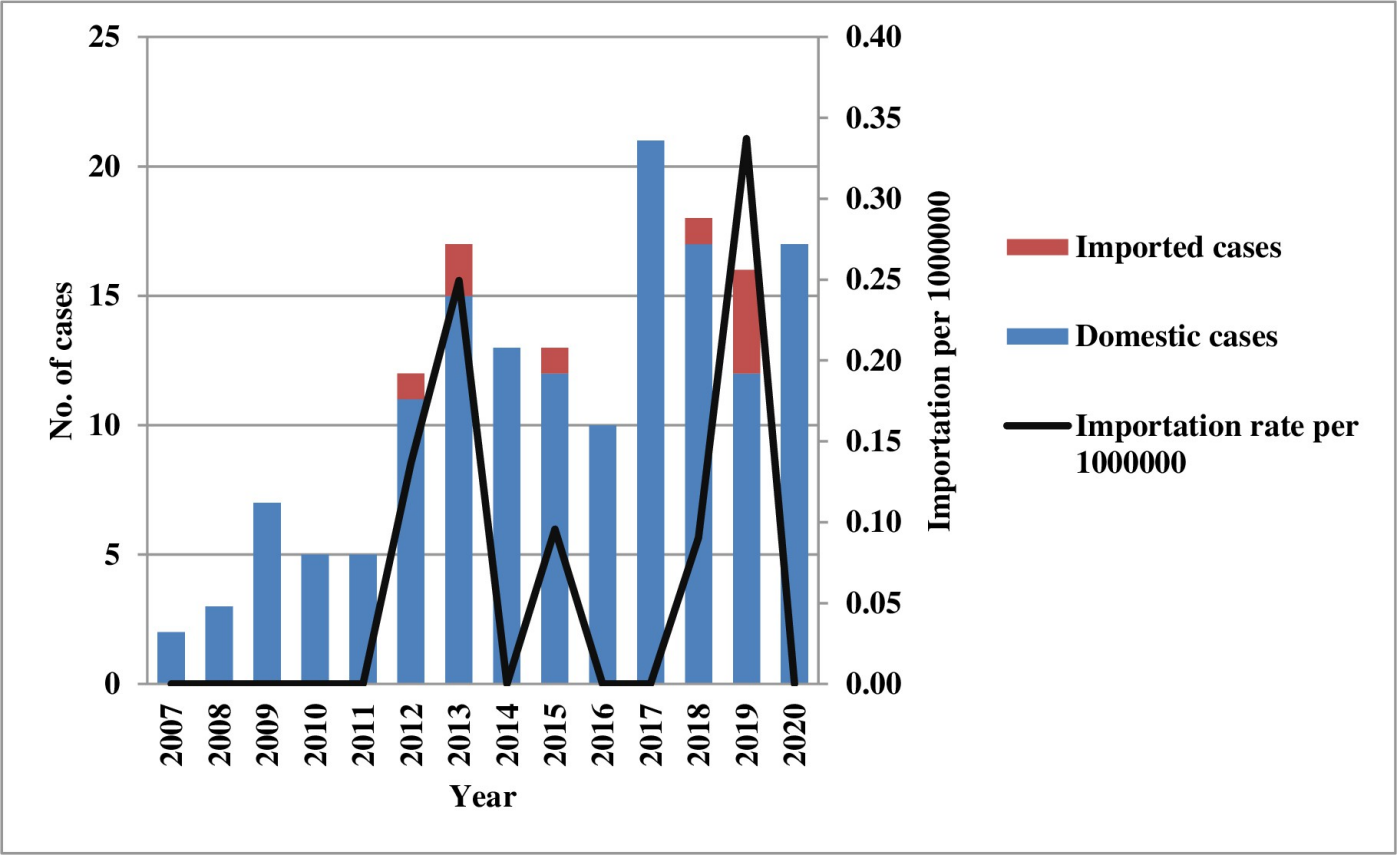
Domestic and imported cases of confirmed toxoplasmosis among patients in Taiwan by year from 2007 to 2020.

**Fig 2:**
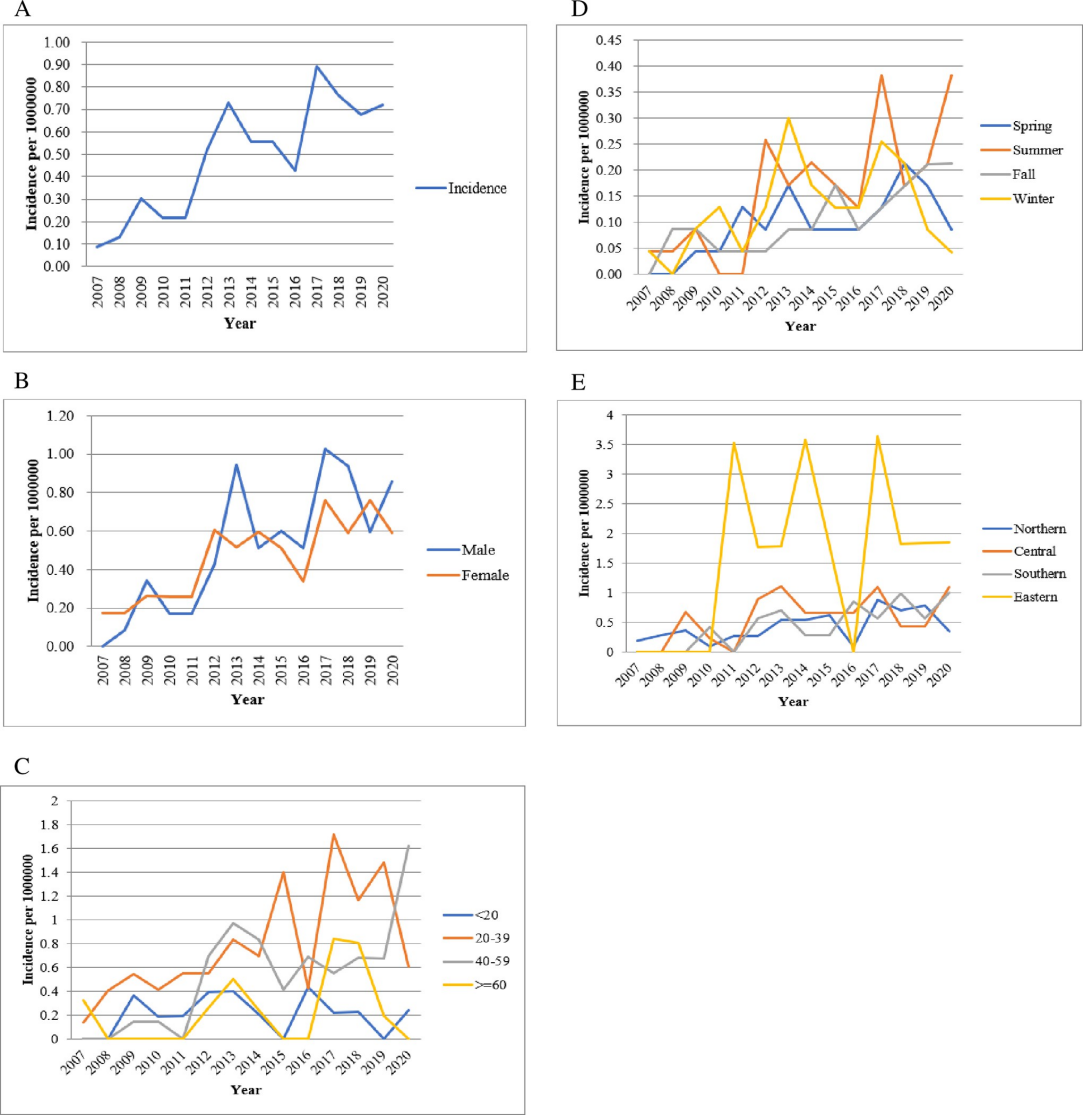
Incidence of confirmed toxoplasmosis among patients in Taiwan according to (A) population, (B) gender, (C) age, (D) season and (E) residency by year from 2007 to 2020.

The annual distribution of the number of confirmed cases of imported infection locally and abroad during 2007–2020 and the import rate (imports per 1,000,000) are shown in Fig 1. The incidence of confirmed cases of toxoplasmosis per million population was 0.09–0.89 (Fig 2A) and 0–0.94 in men during 2007–2020 (Fig 2B). The incidence of confirmed toxoplasmosis cases per million among the 20–39 age group during 2007–2020 was 0.13–1.72 (Fig 2C), the summer was 0–0.38 (Fig 2D), and northern region was 0.09–0.88 (Fig 2E).

## Discussion

Toxoplasmosis is a zoonotic parasitic disease that occurs widely in domestic, wild animals and human [3]. However, underdeveloped countries had a higher incidence than did developed countries [30]. Perhaps the low incidence is due to screening programs or correct diagnosis. Taiwan CDC has cooperated with the blood donation center to collect serum and blood samples from healthy blood donors in Taiwan year by year for diagnosis and screening of toxoplasmosis in order to understand the epidemiological status of toxoplasmosis in Taiwan. This study inferred that the overall medical quality in Taiwan has reached that of developed countries, resulting in a lower incidence of confirmed toxoplasmosis cases. However, the number of cases in Taiwan increased annually during the survey period. During 2007–2011, 2012–2016, 2017–2020 the annual average was 4.4, 13,and 18 cases, respectively. This shows that *T*. *gondii* infection is still an epidemic in Taiwan. In addition to the increasing cost of clinical medical treatment and burden of medical manpower, health threats to people are increasing daily. Clinical and public health experts in Taiwan’s official health departments should put forward effective epidemic prevention policies to control the rising trend of cases.

Although the results showed that the incidence in males was the highest, differences were not observed in the sex of those infected with *T*. *gondii* in our study. Some studies have pointed out that in the thirty-five cases of toxoplasmosis, 18 male patients (51.4%) were affected, with a sex ratio of 1.05(no statistically significant difference) [31].A previous study [32] has shown that children had limited exposure to *T*. *gondii* and that seroprevalence in male and female patients did not differ between those aged 45 years-old and younger. Other studies have reported an overall seropositivity of 21.8% for IgG and 10.3% for IgM antibodies with no sex differences [33]. In terms of age of confirmed cases, some studies showed that the age range of 20–40 years-old was most affected by ocular toxoplasmosis (range:11–65 years-old), with a mean age of 27.2 [34], and the highest infection rate was in the age group of 20–40 years-old (7.6%) [35]. This study showed that the number of cases and incidence in patients aged 20–39 years-old was the highest, similar to previous literature.

Toxoplasmosis has no obvious seasonality and generally occurs during the hot summer and autumn seasons (June–September).A previous study showed that *T*. *gondii* seroprevalence was higher in the summer (37.4%) and autumn (34.9%) than in the spring (24.6%) and winter (23.9%) [36].This finding was similar to that of the present study. The exception in another study indicated that the incidence of acute toxoplasmosis during winter–spring was also significantly higher than that during summer–autumn [37]. This study demonstrated that toxoplasmosis was prevalent in our communities regardless of the month or season, posing a serious threat to health and necessitating constant precautions.

The eastern part of Taiwan includes Hualien and Taitung counties. It faces the vast Pacific Ocean in the east and central mountains in the west. It has magnificent mountainous scenery and rich ecological resources. It is a must-see tourist attraction for many domestic and foreign tourists (the Taroko National Park) [38]. However, the geographical feature of this area comprises more mountains and hills than do flat areas. As such, living and medical conveniences are not as developed as those in the western metropolitan area of Taiwan [39]. Hence, there are fewer medical institutions and personnel in the eastern areas. Medical resources are relatively poor, and chronic and zoonotic infectious diseases (e.g., tuberculosis and scrub typhus) are common, which is a long-standing public health problem [40, 41]. This study is the first to report a high incidence of toxoplasmosis in the eastern region of Taiwan. Although the number of toxoplasmosis cases was the highest in the northern region, the eastern region had the highest incidence rate, which showed that the disease burden in the place of residence should not only be determined by the number of cases, but also by the population susceptible to the disease. The degree of influence of the number of cases truly reflected the geographical invasiveness of the disease. In addition, when comparing the age groups by residential area in this study, the risk of the disease was lower in the 40–59 years-old population living in the northern region, but higher in the 40–59 years-old group in the southern region. Therefore, this study inferred that the geographical distribution of toxoplasmosis in Taiwan was regional, and indicated that the high risk of toxoplasmosis infection in patients in different places of residence varied significantly among different age groups. These warning signs are worthy of attention from clinical and public health experts. We recommend adjusting epidemic prevention measures in a timely manner according to local conditions and responding as soon as possible.

Because of the convenience of transportation (by airplane) when traveling across countries, the time it takes for humans to travel between countries is shorter than the incubation period of most infectious diseases, and pathogens easily spread through travelers [41]. A previous study showed that the prevalence of infection with *T*. *gondii* as well as its genetic structure varied geographically. Therefore, traveling may be considered a risk factor for acquiring the infection [42]. Moreover, another study found that severe toxoplasmosis was imported from tropical Africa to immunocompetent patients in France [43]. This shows that toxoplasmosis is not only a foodborne disease, but also a travel-related illness. Taiwan and neighboring countries such as Southeast Asia, North America, and Europe have very close and frequent exchanges in terms of tourism, business, and work. At present, the total number of foreign workers in Taiwan is approximately 450,000, and most of them are from Southeast Asian countries. The number of spouses of foreign nationals is approximately 500,000, and most of them hail from Southeast Asian countries or China [44]. The epidemic of zoonotic infectious diseases in Taiwan’s neighboring countries is severe. Regarding *T*. *gondii*, large-scale Toxoplasma disease outbreaks have frequently occurred in Asian countries in recent years [45]. In addition, our citizens frequently travel to Europe, America, and Southeast Asia, which increases the risk of contact with pathogens [46]. According to the findings of our survey, there was one case from New Zealand in 2012, one from Singapore in 2013, one from Canada in 2015, one from Singapore in 2018, and one from France, Japan, Vietnam, and Indonesia in 2019. Cases tend to increase annually, bringing a significant disease burden to Taiwan’s border epidemic prevention control. This seriously threatens the health of the Taiwanese people. Therefore, this study suggests that the risk of imported zoonotic infectious diseases from China, Southeast Asia, Europe, and America will continue to exist in the future and may cause local epidemics in Taiwan. We recommend that the health departments of various countries and Taiwan include guidelines for epidemic-prevention measures.

Previous study [47] demonstrates that multiple linear regression analysis shows positive associations between NO2 concentration and amebiasis cases (B value = 2.569, *p* = 0.019), O3 concentration and amebiasis cases (B value = 0.294, *p* = 0.008), and temperature and amebiasis cases (B value = 1.096, *p* = 0.046). Toxoplasmosis and amebiasis cases are both foodborne diseases. Therefore, this study inferred air pollution might contributes to rising incidence of toxoplasmosis. The thermal power generation capacity of Taiwan’s power system (coal, natural gas, heavy oil, and light diesel) accounted for 80.22%, and the number of motor vehicles in Taiwan was 22,297,000 as of December 2020 [48]. These fixed and nonfixed pollution sources often diffuse soot and air pollutants. Polluted air contains particles with diameter of 2.5 microns (PM2.5) or smaller. They are emitted when coal is burned by power plants. The concentration of suspended particulates (PM2.5) generally exceeds 12 micrograms per cubic meter, which pollutes the environment. When PM 2.5 exceeds 35.5 micrograms per cubic meter, the health of sensitive populations (such as adults and children with heart, respiratory, and cardiovascular diseases) will be affected [49]. Some fields even reach hundreds of micrograms per cubic meter, which has reached "unhealthy" levels in the air quality index of the Taiwan EPA. This poses a serious threat to human health. Because PM2.5 is very small, it is easy to inhale deep into the lungs, causing short-term respiratory problems. Several studies have shown that the long-term inhalation of these particles aggravates asthma attacks, leading to chronic obstructive pulmonary diseases, cardiovascular disease, low birth weight in newborns, and other problems [50]. Air pollution appears to be the biggest environmental risk factor for the deaths in people in the United States and other developed countries. Previous studies have focused on the impact of various meteorological factors on hand-foot-mouth disease (HFMD) but do not know how individual foods or the entire eating process or behavior affect human health through poor air quality [51]. Our study is similar to that of HFMD in terms of pathogen transmission, both of which are caused by contact with and ingestion of food or water containing pathogens.

Previous findings show that community-level agricultural, environmental, and socioeconomic factors may be important with regard to the rates of foodborne diseases, such as *Salmonella typhimurium* infection [23]. A previous study [52] indicated that warmer temperatures are expected to increase the risk of food-borne pathogens, water-borne diseases, and vector-borne zoonosis in human and animal populations. A previous study [53] also indicated that high levels of atmospheric CO2 emissions and toxic pollutants in air, water, and food have serious repercussions on all life systems, including living beings, the environment, and economy. Therefore, this study aimed to assess the impacts of climatic factors and air pollutants on *T*. *gondii* cases in Taiwan. However, few studies have evaluated the effects of climate and air pollution on toxoplasmosis incidence. This study confirms that the concentration of atmospheric fine particulate matter (PM2.5) has a negative impact on human health and positively correlates with the increase in *T*.*gondii* cases by increasing the concentration of PM2.5. It was determined that for every 1 ug/m^3^ increase in the concentration of PM2.5, the number of *T*.*gondii* cases increased by 0.095. Therefore, this study suggests that government environmental protection departments, enterprises, and the public should actively improve air quality, reduce the concentration of air pollutants (particularly PM 2.5), and change diet, culture, or habits to effectively reduce the impact of air pollutants on health. The results of this study can be used as a reference to develop early warning systems and specific intervention measures for vulnerable groups. CO is one of the primary components of emissions from light-duty vehicles and comprises 77% of all pollutants emitted in terms of their concentration. A previous study found that diastolic blood pressure decreases with increasing CO concentration after exposure [54]. This study showed that there was also a significant negative correlation between CO and the number of *T*. *gondii* cases when other air pollution environmental factors are considered; that is, when the concentration of CO increased, the number of *T*.*gondii* cases decreased. This study is the first to describe the assessment of environmental factors (PM 2.5, CO concentration) on the number of *T*.*gondii* cases and inferred that changes in the concentration of environmental factors will affect the number of human toxoplasmosis cases. However, this study only traces air pollution data for eight years. Further studies should expand monitoring data and collect more massive amounts of information to further determine the impact of other environmental exposure factors (risk factors) on toxoplasmosis.

This study has two limitations. First, the statistical data on infectious diseases published by the CDC in Taiwan online only provide basic epidemiological data on patients with toxoplasmosis without clinical data. Therefore, this study could not compare the differences or trends in clinical data or symptoms. Because Taiwan has not a high incidence of *T*. *gondii* infection, there is no routine screening program for congenital toxoplasmosis (CT) in Taiwan. However, we couldn’t rule out the under diagnosis of the *T*. *gondii* infection. Second, no information regarding the genotype of *T*.*gondii* has been published on this platform. Hence, this study could not determine the genotype that is prevalent in Taiwan. In addition, the genetic relationship of *T*.*gondii* genotypes between Taiwan and other countries could not be determined. This study has one advantage: the information from existing public network platforms in Taiwan is timely and accurate. Moreover, the platform keeps data for many years so that researchers or institutions can study the statistics or applications of monitored infectious disease data.

## Conclusions

This study is the first to report the epidemiological characteristics and trends of toxoplasmosis cases in Taiwan between 2007 and 2020. In 2017, toxoplasmosis had the highest confirmed case rate (0.89 per million people). In recent years, the risk of imported cases from abroad has increased sharply, resulting in a burden of disease, public health challenges, and epidemic prevention. The geographical distribution of toxoplasmosis in Taiwan is regional in nature. The risk of toxoplasmosis among patients living in different areas increases with age. The change in the exposure concentration of some environmental factors (PM 2.5, CO) will affect the increase or decrease in toxoplasmosis cases. This information will be useful for policy-makers and clinical experts to direct prevention and control activities for wildlife, livestock, and pet animals infected with *T*. *gondii* which causes the most severe illness and the greatest burden to Taiwan. Important data have been identified to inform future surveillance and research in Taiwan.
